# Research on Tin Bath Bottom Bricks for Float Glass Furnaces

**DOI:** 10.3390/ma19101989

**Published:** 2026-05-11

**Authors:** Kuiqing Guo, Benjun Cheng, Weibin Xu, Xiaocheng Liang, Liming Zou, Wencheng Wang, Guoqi Liu

**Affiliations:** 1 School of Energy Science and Engineering, Central South University, Changsha 410083, China; 253912037@csu.edu.cn (K.G.); 232912061@csu.edu.cn (W.X.); 253911025@csu.edu.cn (L.Z.); 253912062@csu.edu.cn (W.W.); 2 School of Materials Science and Engineering, Shanghai University, Shanghai 200444, China; lxc2025@shu.edu.cn (X.L.); liuguoqi@shu.edu.cn (G.L.); 3 State Key Laboratory of Advanced Refractories, Shanghai University, Shanghai 200444, China

**Keywords:** mullite, low-cement castable, thermal shock resistance, mechanical properties, float glass furnace

## Abstract

The bottom brick is a critical component of float glass furnace tin baths, serving under harsh conditions including high temperature, tin penetration, hydrogen diffusion and alkali attack. Traditional flint clay-based bottom bricks suffer from high porosity and insufficient service performance. In this study, a high-performance low-cement castable was developed by introducing mullite aggregates to partially replace flint clay. The effects of mullite particle size and addition content on sintering behavior, mechanical properties, thermal shock resistance, refractoriness under load and hydrogen diffusion were systematically investigated. The results demonstrate that, compared with the existing tin bath bottom bricks applied in float glass furnaces, the introduction of 18 wt% mullite with a particle size of 5–3 mm can significantly increase the bulk density, reduce the apparent porosity, enhance the mechanical strength at both room temperature and high temperature, and achieve a higher refractoriness under load and lower hydrogen diffusion capacity. Accordingly, a novel tin bath bottom brick with excellent comprehensive properties for float glass furnaces was successfully developed.

## 1. Introduction

The tin bath, as the core of glass production, is one of the three major examples of thermal equipment in the float glass manufacturing process. Serving as the glass-forming zone, its main body is a trough-shaped shell structure constructed from refractory materials and containing molten tin [1,2]. The tin bath bottom brick constitutes the refractory lining of this trough that holds the molten tin. High-temperature molten glass flows from the canal into the tin bath containing molten tin, where it spreads, stretches, and cools on the surface of the tin to form a glass ribbon of uniform thickness. Throughout the production process, the operating temperature of the tin bath typically ranges from 1100 °C to 600 °C [3]. The molten tin above the bottom bricks can penetrate inwards through the pores within the bricks. If penetrating pores exist, the anchor bolts at the bottom of the bath are highly susceptible to corrosion, leading to the floating of the bottom bricks. During production, the tin bath is filled with protective gas. Hydrogen molecules, due to their small size, possess strong permeability. Hydrogen dissolves in the molten tin and diffuses into the bricks through their pores. However, if the bottom bricks undergo changes, particularly temperature increases, gas can precipitate outwards from within the bricks, passing through the molten tin and creating bubbles on the lower surface of the glass ribbon. Simultaneously, alkaline oxides or alkaline ingredients from the molten glass permeate and diffuse through the tin to the surface of the bottom bricks, undergoing a nepheline-forming reaction with the brick material. This reaction leads to the spalling of the bottom bricks [4,5]. Therefore, the requirements for tin bath bottom bricks are not only low porosity and high thermal shock resistance but also strong alkali corrosion resistance to enhance their service life. Nevertheless, in the current float glass industry, traditional tin bath bottom bricks prepared with flint clay without mullite addition exhibit a high apparent porosity ranging from 18% to 20%. Accordingly, it is urgent to develop a novel mullite-reinforced low-cement castable suitable for the severe service conditions of tin baths. Low-cement castables are defined as refractory castables with the addition of calcium aluminate cement of 2–6% (significantly lower than the 10–20% cement dosage of traditional castables) and a CaO content controlled within 1.0–2.5%. This type of castable relies on ultra-fine powders, including silica fume and α-Al_2_O_3_, high-efficiency water reducers, and close packing gradation design to achieve low water demand, low porosity, high bulk density, and excellent high-temperature mechanical strengthening performance. In terms of apparent porosity, low-cement castables range from 12% to 18%, while traditional castables are between 20% and 30%, and zero-cement castables maintain a low level of 10–15%. In terms of bulk density, the values of low-cement castables are 2.6–3.0 g/cm^3^, traditional castables are 2.2–2.5 g/cm^3^, and zero-cement castables reach 2.8–3.2 g/cm^3^. In terms of permanent linear change, low-cement castables show a small fluctuation range of ±0.5%, traditional castables are ±1.0%, and zero-cement castables are only ±0.3%. In terms of maximum service temperature, low-cement castables can withstand 1450–1600 °C, traditional castables are limited to 1300–1400 °C, and zero-cement castables can reach 1600–1800 °C. In terms of cold crushing strength tested at 110 °C, low-cement castables achieve 60–100 MPa, traditional castables are 30–50 MPa, and zero-cement castables attain 80–120 MPa. In terms of high-temperature crushing strength tested at 1400 °C, low-cement castables range from 70 to 90 MPa, traditional castables are only 20–40 MPa, and zero-cement castables reach 90–130 MPa. In terms of hot modulus of rupture tested at 1200 °C, low-cement castables are 15–25 MPa, traditional castables are 5–10 MPa, and zero-cement castables are 20–35 MPa [6].

Currently, research on the tin bath primarily focuses on the simulation of the temperature field and flow field of the glass flowing through it, as well as analyses of tin bath structural design, all aimed at improving glass quality. Li Luyao et al. [7] investigated the geometry of the tin bath entrance by altering the width of the working back refractory and the angle between the working back refractory and the restraint blocks, providing guidance for tin bath inlet design. Li Luyao et al. [8] conducted theoretical and experimental studies on the hydrodynamic and thermal behavior of molten tin to verify its potential impact on glass output. In recent years, research on the chemical composition and microstructure of tin bath bottom bricks has been limited. Therefore, it is necessary to conduct such studies to enhance their high-temperature properties, such as thermal shock resistance and corrosion resistance, thereby providing the float glass industry with a more economically efficient, long-lasting service material that ensures high product quality.

Mullite is a mineral phase belonging to the silicate chain, with an orthorhombic crystal structure. It typically exists in prismatic or acicular forms and has a melting point exceeding 1800°C. [9] It can be incorporated into castables either directly as aggregates or added as fine powder to the matrix. If a mullite network structure is formed at high temperatures from fine aluminum–silicate raw materials, it can strengthen the bonding interface between the aggregates and the matrix [10,11,12]. Refractory products manufactured using mullite as a raw material exhibit excellent high-temperature resistance, thermal shock stability, and corrosion resistance, making it a high-quality refractory. Its maximum service temperature can range between 1500°C and 1700°C [13,14]. Furthermore, it maintains outstanding structural integrity and mechanical strength under high-temperature environments and demonstrates good resistance to erosion by alkaline chemical media.

Gao Hui et al. [15] investigated the effect of silica fume content on mullite castables using mullite aggregates of different particle sizes. Mai Haixiang et al. [16] explored the influence of mullite on the Al_2_O_3_/SiO_2_ ratio in the matrix on the properties of high-bauxite–mullite castables. Sharma R. et al. [17] studied the mechanical properties of low-cement castables containing mullite aggregates synthesized from sillimanite. Wang Cankun et al. [18] examined the effects of different types of sintered mullite aggregates on the properties of corundum–mullite castables. Derensy M. et al. [19] investigated the impact of nano-scale additives on the performance of corundum–mullite castables. The aforementioned studies indicate that the performance of mullite castables can primarily be improved by adjusting the particle size distribution, particle size composition, and the types of matrix and aggregates [20,21,22,23]. Through these modifications, the properties of mullite castables have been optimized.

Currently, although commercial bottom bricks primarily based on sintered flint clay offer lower costs, their long-term durability and resistance to molten glass penetration still require improvement. In this study, industrial flint clay was used as the matrix. By adding mullite with different contents and particle sizes, the effects of the mullite variable on the mechanical properties, thermal shock resistance, and the microstructure of the material were investigated. This research aims to elucidate the mechanism of the influence on the low-cement castable by mullite, which enhances the performance and microstructural evolution. The findings are expected to provide a theoretical basis and reliable experimental data to support the formulation design of high-performance, long-life tin bath bottom bricks, thereby enabling the preparation of a novel mullite-reinforced low-cement castable for this application.

However, most current studies on the tin bath are focused on the simulation of temperature field, flow field and structural design, while systematic research on the composition, microstructure and service performance of tin bath bottom bricks remains insufficient. Traditional tin bath bottom bricks prepared with flint clay exhibit high apparent porosity, insufficient resistance to molten tin penetration, hydrogen diffusion and alkali corrosion, which lead to structural degradation, shortened service life and quality defects of glass products. Although mullite has been widely applied in refractory castables, existing studies mainly concentrate on performance improvement for metallurgical and cement industries, lacking targeted investigation on key properties required under the harsh service conditions of float glass tin baths, such as low hydrogen permeability, excellent alkali corrosion resistance and high thermal shock stability. In addition, the quantitative regulation mechanism of mullite particle size and its addition content on the sintering behavior, mechanical properties and microstructural evolution of low-cement castables for tin bath bottom bricks has not been systematically revealed, and a clear optimal formulation and engineering application basis are still absent. Therefore, in this work, a low-cement castable for tin bath bottom brick was taken as the research object, and mullite aggregates were used to partially replace traditional flint clay. The effects of mullite particle size and its addition on bulk density, apparent porosity, room- and high-temperature mechanical strength, refractoriness under load, hydrogen diffusion performance and thermal shock resistance were systematically investigated, aiming to provide experimental data and theoretical support for the development and engineering application of high-performance and long-life tin bath bottom bricks.

## 2. Experiment

### 2.1. Raw Materials

The raw materials used in the experiment include flint clay (Al_2_O_3_ > 46 wt%), mullite (Al_2_O_3_ > 70 wt%), silica fume (average particle size 0.15 μm, SiO_2_ > 95 wt%), α-Al_2_O_3_ micropowder (Al_2_O_3_ > 99 wt%, 5 μm), high-alumina powder (200 mesh), water reducer, and calcium aluminate cement (69.4 wt%Al_2_O_3_, 29.6 wt% CaO). The formulation compositions of the test specimens are shown in Table 1. Since the bulk density of mullite is higher than that of flint clay, increasing the addition of mullite in the combined aggregates reduces the total volume of the aggregates for a given total weight. Consequently, the volume proportion between the aggregate particles and the fine powders, as well as their packing state, changes. To accommodate this change in the volumetric proportion of the aggregates, the addition of silica fume, cement, and α-Al_2_O_3_ micropowder in the formulation should be appropriately adjusted. All the aforementioned raw materials were sourced from Luoyang Hexin Refractories Co., Ltd., Luoyang City, Henan Province, China. 

### 2.2. Methods and Preparation

The raw materials were uniformly mixed according to the proportions shown in Table 1, then poured into a mixer and dry-mixed for 3–5 min. Subsequently, an appropriate amount of water was added, followed by another 5 min of mixing. The mold was placed on a vibrating table, and the vibration table was started. The mixed castable was poured into the mold and vibrated to achieve forming, with a general vibration time of 60–120 s. After forming, the specimens were cured in the mold at room temperature for one day and then demolded. They were dried in an oven at 110 °C for 12 h, followed by heat treatment at 1160 °C for 3 h. According to Chinese national standards GB/T 3001-2017 [24] and GB/T 5072-2023 [25], specimens with dimensions of 40 mm × 40 mm × 160 mm were prepared for flexural strength (or compressive strength) testing, and specimens of 25 mm × 25 mm × 125 mm were prepared for thermal shock resistance testing. During the thermal shock resistance test, following the Chinese national standard GB/T 30873-2014 [26], the water quenching method was adopted. The specimens were heated to 1100 °C, then rapidly immersed in water at 20 °C, subsequently dried at 110 °C for 24 h, and their flexural strength was measured. 

The residual strength ratio (R_r_) after thermal shock is a vital comprehensive indicator for evaluating the thermal shock resistance and structural durability of refractory materials. It refers to the proportional ratio of the cold flexural strength of specimens retained after applying repeated rapid alternating temperatures to the original strength before thermal shock testing. Under periodic high-temperature heating and rapid cooling cycles in actual furnace operation, refractories with a low residual strength ratio are prone to rapid strength attenuation, crack propagation and eventual structural failure. The residual strength ratio (R_r_) after thermal shock was calculated using Equation (1) to quantify the thermal shock resistance of the material.


(1) $$R_{r}=\frac{\left|R_{1}-R_{0}\right|}{R_{0}}\times 100\%$$

In the equation, R represents the flexural strength, with the subscripts 0 and 1 indicating values before and after the test, respectively.

**Figure 1:**
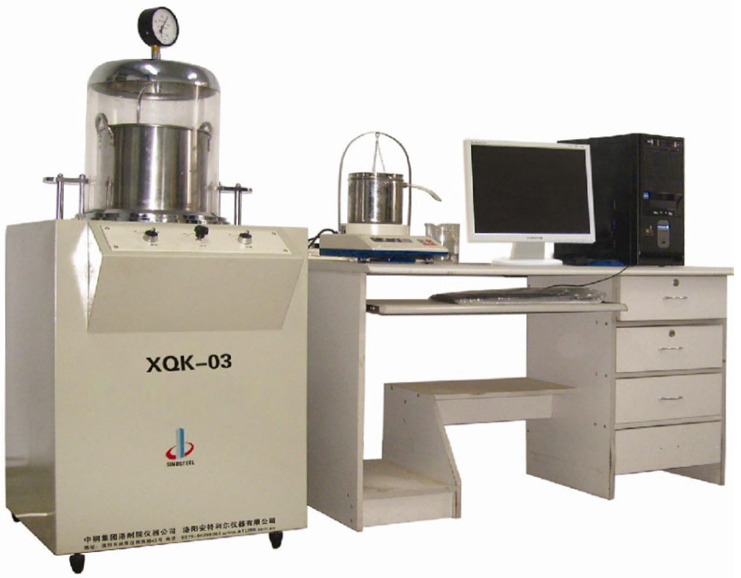
Apparent porosity and bulk density tester.

Apparent porosity and bulk density are fundamental comprehensive indices for characterizing the internal compactness and pore structure of refractory materials. They refer to the basic physical parameters that reflect the proportion of internal open pores and the dense stacking state of the refractory matrix after sample preparation and drying treatment. Under actual high-temperature service environments, excessively high porosity accelerates molten slag penetration and gas erosion, thereby reducing the overall service life and structural stability of refractory linings. According to standard GB/T 2997-2015 [27], the apparent porosity and bulk density tester is shown in Figure 1.

**Figure 2:**
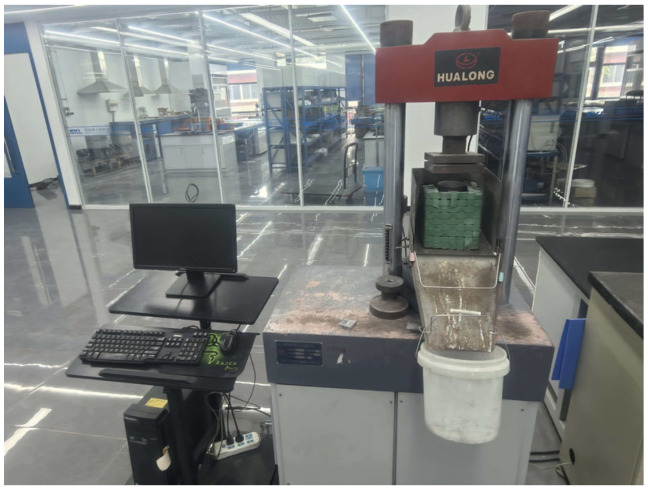
Cold crushing strength testing machine.

Cold crushing strength (CCS) is an essential mechanical indicator for evaluating the room-temperature structural stability and integral bearing capacity of refractory materials. It refers to the maximum compressive load that a refractory specimen can withstand without fracture or irreversible damage under continuous axial compression at room temperature. When exposed to excessive mechanical extrusion and external impact during practical application, refractory components with insufficient cold crushing strength are prone to cracking and structural damage. According to standard GB/T 5072-2023, the cold crushing strength testing machine is shown in Figure 2.

**Figure 3:**
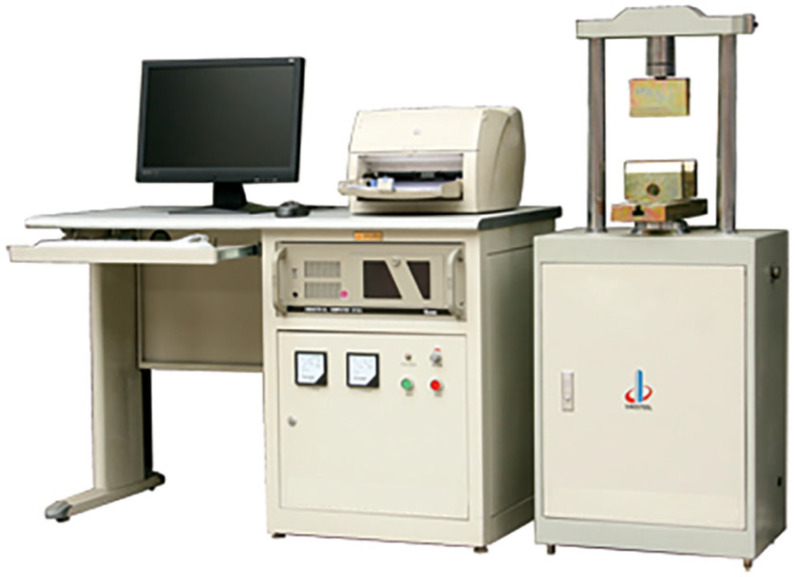
Modulus of rupture testing machine.

Modulus of rupture (MOR) is a key mechanical indicator for assessing the room-temperature flexural resistance and structural integrity of refractory materials. It refers to the critical bending stress that a refractory specimen can endure before fracture occurs under continuous three-point or four-point bending loading at ambient temperature. Under actual industrial operating conditions, refractory linings with low cold modulus of rupture are susceptible to bending deformation, cracking and structural spalling under external mechanical stress and thermal shock. According to standard GB/T 3001-2017, the modulus of the rupture testing machine is shown in Figure 3.

Refractoriness under load (RUL) is a crucial indicator for evaluating the high-temperature structural strength of refractory materials. It refers to the critical temperature at which a refractory product undergoes significant plastic deformation internally when heated to a certain temperature under a constant load. When subjected to a load exceeding this critical value under actual service conditions, the product may sink or collapse. According to standard YB/T 370-2016 [28], this experiment employed the non-differential method to determine the initial softening temperature for dense refractory castables under a load of 0.2 MPa, defined as the temperature at which the specimen is compressed to 0.6% of its original height.

**Figure 4:**
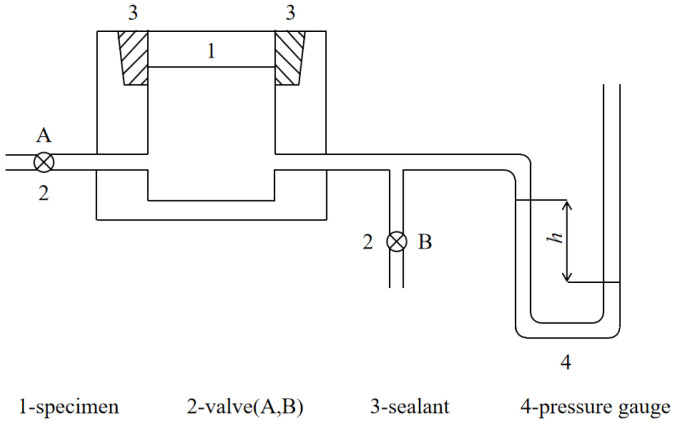
Principle diagram of hydrogen diffusivity tester.

**Figure 5:**
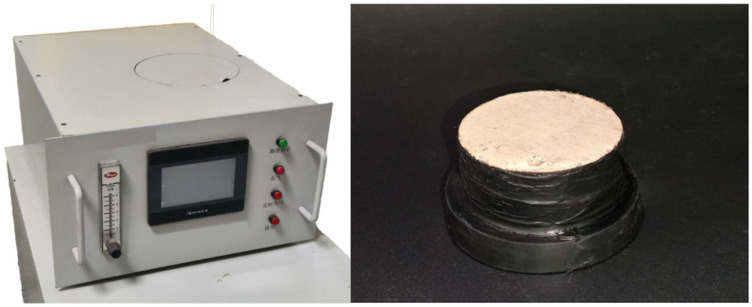
Facilities and test samples of hydrogen diffusion experiment.

The hydrogen diffusion tester measures the diffusion characteristics of hydrogen through a refractory tester based on the differential pressure method. According to standard JC/T 926-2003 [29], the test apparatus is shown in Figure 4, and the facilities and test samples are shown in Figure 5. During the test, dry hydrogen gas is introduced into the test chamber containing the specimen at a certain pressure, with the other end of the chamber open to the atmosphere. After a period of time, the air inside the test chamber is displaced. Subsequently, the valves at both ends of the test chamber are simultaneously closed. The pressure inside the chamber gradually decreases. When it reaches its lowest point and begins to rise again, the corresponding pressure difference relative to the external atmospheric pressure is defined as the hydrogen diffusion.

### 2.3. Characterization

The microstructure of the heat-treated samples (polished surfaces) was observed using field emission scanning electron microscopes (FESEM; MIRA4, TESCAN, Brno, Czech Republic; and JSM-IT800, JEOL, Akishima, Tokyo, Japan), both equipped with energy-dispersive X-ray spectroscopy (EDS) systems. All polished sample surfaces were sputter-coated with gold prior to observation.

## 3. Results and Discussion

### 3.1. Characterization and Functional Analysis of Raw Materials

The incorporation of mullite into the specimens introduces its characteristic orthorhombic crystal structure. Mullite is a stable compound under normal pressure, with the chemical formula 3Al_2_O_3_·2SiO_2_. Upon cooling of the silicate melt, acicular mullite crystals with a linear morphology precipitate. In high-alumina products, these acicular mullite crystals interlock to form a dense mullite skeleton, thereby enhancing the mechanical properties. When high-alumina cement is used as a binder, the inevitable introduction of calcium oxide (CaO) can lead to the formation of low-melting phases during the firing process of the bottom bricks, which compromises their high-temperature performance [30]. Conversely, the addition of α-Al_2_O_3_ micropowder can fill pores and effectively improve the high-temperature properties of the specimens. Silica fume, an amorphous spherical material with good dispersibility, can fill pores and reduce the water demand of the castable. At elevated temperatures, it reacts with α-Al_2_O_3_ to form mullite, further enhancing the high-temperature performance of the castable [31]. The inclusion of a composite water reducer not only decreases the water requirement for the castable but also improves the compactness of the green specimens, improving the mechanical properties of the material.

### 3.2. Thermodynamic Properties

**Figure 6:**
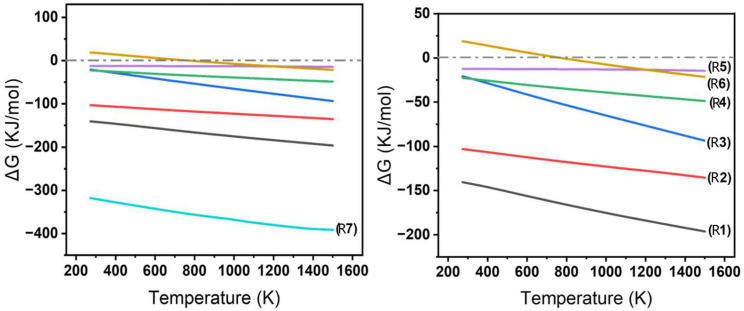
Gibbs free energy–temperature curve.

Regarding the raw materials involved in the experiments, the possible chemical reactions are shown in Table 2, and the relationship between the Gibbs free energy and temperature for these reactions in the range of 298–1443 K is presented in Figure 6. Within the considered temperature range, the Gibbs free energy of most reactions is negative, with the exception of Reaction (6). This reaction only satisfies the thermodynamic conditions for a spontaneous reaction at temperatures around 800 K. The Gibbs free energy of Reaction (7) is significantly lower than that of other reactions, indicating that, under identical thermodynamic conditions, the formation of sodium feldspar is the dominant reaction, and this is the basis of the glass phase.

When the Gibbs free energy of the reaction is less than zero, it indicates that the reaction is thermodynamically favorable and can proceed spontaneously. However, in practical situations, if the kinetic conditions are not satisfied, the reaction may only exhibit a tendency to proceed.

### 3.3. Mechanical Properties

**Figure 7:**
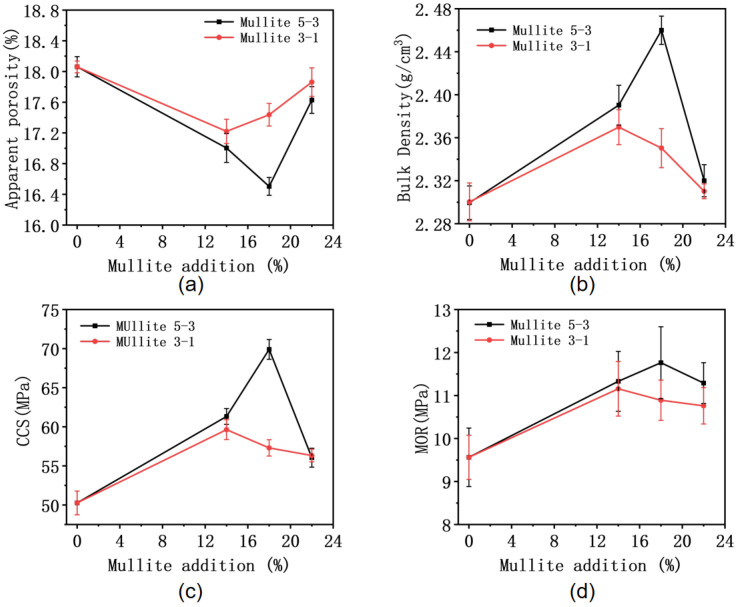
(**a**) Apparent porosity; (**b**) Bulk density; (**c**) Cold crushing strength; (**d**) Modulus of rupture.

The apparent porosity, bulk density, cold crushing strength (CCS), modulus of rupture (MOR) at room temperature, refractoriness under load (RUL), and residual strength ratio after thermal shock were tested for each specimen. The apparent porosity and bulk density are presented in Figure 7a,b, with the corresponding detailed data summarized in Table 3 and Table 4. Based on the original formulation, the addition of mullite led to a decreasing trend in apparent porosity and an increasing trend in bulk density. However, as the addition increased within the mullite (3–1 mm) series, the apparent porosity increased and the bulk density correspondingly decreased. Compared to the (3–1 mm) series, the incorporation of mullite (5–3 mm) was more effective in reducing the apparent porosity and increasing the bulk density of the refractory specimens, indicating that the (5–3 mm) mullite series is more helpful in forming a material structure with higher density. When the addition of the mullite (5–3 mm) series reached 18 wt%, the specimen achieved its maximum bulk density and minimum apparent porosity, indicating optimal densification at this point.

The mechanical properties at room temperature are shown in Figure 7c,d. The corresponding data of cold crushing strength and modulus of rupture are listed in Table 5 and Table 6, respectively. The addition of mullite also positively enhanced the mechanical properties of the refractory material. Although the mullite (5–3 mm) series effectively improved the CCS and MOR, excessive addition diminished the mechanical properties. While the mechanical properties of the mullite (3–1 mm) series were generally improved compared to the specimen without mullite, the degree of improvement was less than that observed for the (5–3 mm) series. This is because the cement mineral phase generates high-activity alumina at approximately 600 °C. Part of the as-produced active alumina can react with silica fume to form mullite, thereby facilitating the sintering process and improving the comprehensive performance of materials. For the two mullite aggregate particle size refractories, the room-temperature MOR of the refractories showed little difference as the addition increased. However, in the CCS test, the specimen with 18 wt% addition of the (5–3 mm) mullite series reached its peak; by contrast, all specimens in the (3–1 mm) mullite series exhibited values below this peak and remained within a relatively stable range.

**Figure 8:**
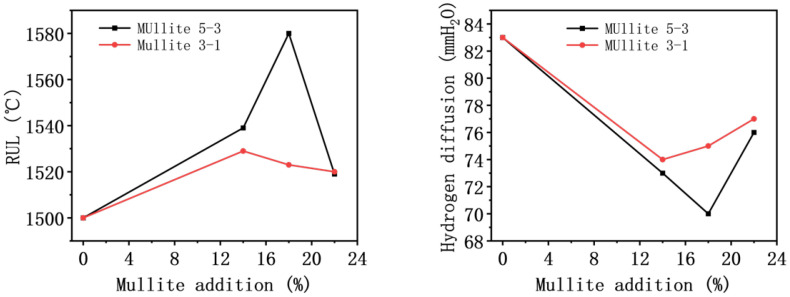
Refractoriness under load (RUL) and hydrogen diffusion of samples.

Based on the aforementioned experiments, the tin bath bottom brick was prepared by appropriately adjusting the other raw material ratios and additives. It can be concluded that the optimal formulation involves the addition of 18 wt% mullite (5–3 mm) aggregates. After testing, the refractoriness under load (RUL) for this formulation was 130 °C higher than that of the brick without mullite addition, reaching 1580 °C. Furthermore, the hydrogen diffusion decreased by 75 mmH_2_O, achieving a value of 70 mmH_2_O. The detailed data of refractoriness under load and hydrogen diffusion are presented in Table 7 and Table 8, respectively, and the relevant data are plotted in Figure 8.

**Figure 9:**
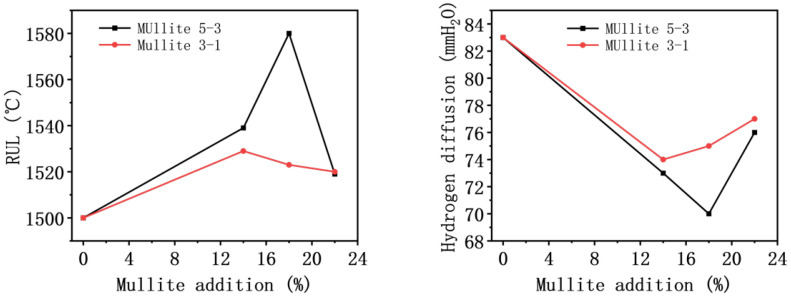
Mechanical resistance of sintered samples at high temperature.

During the thermal shock tests, the flexural strength of all castable specimens gradually decreased as the number of thermal shock cycles increased. As shown in Table 9 and Figure 9, after one thermal shock cycle, the flexural strength of all castables decreased sharply. After three and five thermal shock cycles, the decrease in flexural strength for all castables gradually leveled off. After ten thermal shock cycles, the flexural strength of all specimens decreased again, with this final decrease being the most pronounced.

### 3.4. Microstructural Analysis

**Figure 10:**
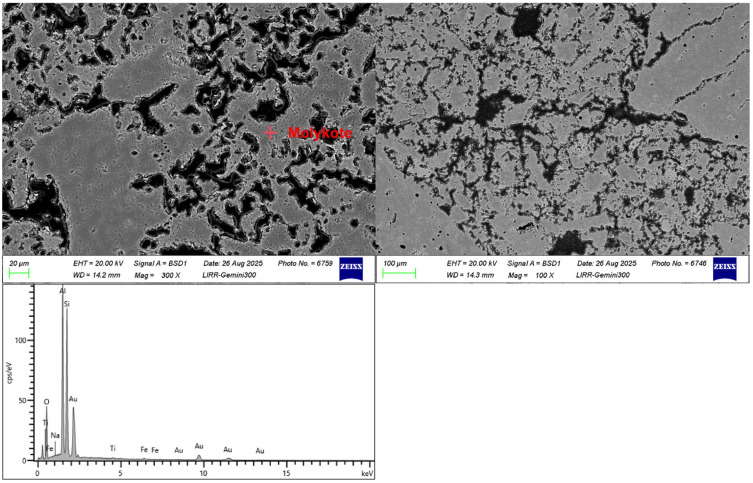
Microstructure and energy-dispersive spectra of tin bath bottom bricks.

As shown in Figure 10, the bonding between the aggregates and the matrix is satisfactory, with microcracks present in some areas. These microcracks are beneficial for improving the thermal shock resistance of the material. The fine powders within the matrix are well-bonded and relatively uniformly distributed. There is a higher proportion of closed pores, which is the primary reason for the low hydrogen diffusion rate. Energy-dispersive spectroscopy (EDS) analysis revealed that in the distinctly dark gray regions of the matrix, the Al_2_O_3_ content is 43.92 wt.% and the SiO_2_ content is 54.44 wt.%, indicating that the main mineral phase in the matrix is Molykote. This is one of the reasons for the material having good high-temperature performance.

## 4. Analysis

Based on the aforementioned results, it can be observed that the specimens incorporating 5–3 mm mullite exhibited the optimal performance in terms of sintering behavior, mechanical properties, and thermal shock resistance. Increasing the addition of mullite in the combined aggregates reduces the total volume percentage occupied by the aggregate particles; consequently, the amount of fine powder surrounding the aggregate particles increases, which can enhance the internal bonding strength of the material. However, if the mullite content in the aggregates becomes excessively high, it leads to an excessive volume fraction of fine powders. This results in a decrease in the bulk density of the specimens, reduced densification, and increased porosity, consequently leading to a decline in physical properties at room temperature, with both the flexural strength and compressive strength exhibiting a trend of first increasing and then decreasing. The binding phases of the formed tin bath bottom bricks are primarily CAH_10_, C_2_AH_8_ or C_3_AH_6_. The heat treatment temperature for these bricks is typically around 1200 °C. During the heating process of this heat treatment, amorphous Al_2_O_3_ forms at approximately 600 °C. This Al_2_O_3_ possesses high reactivity and preferentially reacts with silica fumes and impurities in the raw materials to form a glassy phase, which promotes the sintering process. The surplus Al_2_O_3_ reacts with silica fume to form mullite, a reaction accompanied by a volume expansion, which reduces the linear shrinkage of the material.

Mullite possesses excellent mechanical properties and high-temperature performance. Increasing the addition of mullite is beneficial for enhancing the high-temperature properties of the material. Utilizing mullite with a larger particle size as the aggregate can not only improve the high-temperature strength of the material but also increase its refractoriness under load (RUL) [32]. The distinct difference in thermal expansion coefficients between mullite particles and flint clay particles, with the linear thermal expansion coefficient of mullite ranging from $4.5\sim 5.5\times 10^{-6}{\;{}^{\circ}C}^{-1}$ and that of flint clay ranging from $4.5\sim 6.0\times 10^{-6}{\;{}^{\circ}C}^{-1}$, facilitates the formation of microcracks during heating and long-term high-temperature service conditions [33]. These microcracks can provide a certain buffer zone within the material, leading to microcrack toughening, which further improves the thermal shock resistance of the material. However, if the mullite addition becomes excessive, it can cause a decrease in the material’s densification and strength, thereby adversely affecting its performance. Concurrently, through the optimization of raw material matching and the improvement of additives, the densification and sintering strength of the material were further enhanced, while the porosity and hydrogen diffusion were reduced.

After one thermal shock cycle, the flexural strength of the castables decreased sharply, which is attributed to the glassy phase present in the material matrix. After three and five thermal shock cycles, the decrease in flexural strength for all castables gradually leveled off, indicating that the impact of thermal shock on the castables was relatively minor during this stage, with a slower rate of crack propagation. After ten thermal shock cycles, the flexural strength of all specimens decreased again, with this final decrease being the most pronounced. This suggests that the strength of the material diminished to a critical threshold, where it was insufficient to withstand the thermal stresses generated by the thermal shock, leading to another sudden drop in the flexural strength of the castables.

## 5. Conclusions

An experimental investigation was conducted to compare the performance and evaluate the engineering applicability of aluminosilicate castable specimens based on variations in mullite particle size and addition content. The service properties of the specimens, including sintering behavior, mechanical properties, and thermal shock resistance, were tested. The specimens were characterized using techniques such as SEM and EDS. The results are as follows:

(1) The incorporation of mullite aggregates, particularly with a particle size of 5–3 mm and an optimal content of 18 wt.%, significantly improves the sintering behavior, mechanical properties, and thermal shock resistance of the castables. The apparent porosity of the specimens decreases, while the bulk density increases, thereby enhancing the modulus of rupture and cold crushing strength at room temperature.
(2) The addition of mullite, coupled with the optimized matrix, creates a synergistic effect that contributes to improved structural densification, thereby enhancing the mechanical properties at both room and high temperatures. The presence of mullite also promotes a microcrack toughening mechanism, which improves thermal shock resistance.
(3) The thermal shock tests indicate that the significant decrease in residual strength after one cycle is attributed to the glassy phase present in the material matrix. The stabilization of residual strength observed during subsequent cycles is primarily due to the incorporation of mullite aggregates and the optimization of the matrix, which enhanced the material’s thermal shock resistance. After ten cycles, the strength of the material diminished to a critical threshold, where it was insufficient to withstand the thermal stresses generated by the thermal shock, consequently leading to another sudden drop in the flexural strength of the castables after thermal shock.

Future research will be carried out in the following aspects. Firstly, the corrosion kinetics and microstructural evolution of the prepared mullite-reinforced low-cement castables under the attack of alkali vapor and molten tin will be further studied, so as to clarify the inhibition mechanism of mullite on nepheline formation and tin penetration. Secondly, the long-cycle thermal shock resistance, high-temperature creep behavior and mechanical fatigue performance of the material will be evaluated to establish a reasonable service life prediction model for tin bath bottom bricks. Thirdly, the material formulation will be further optimized by introducing nano-scale powders or composite aggregates, and ultra-low-cement or cement-free bonding systems will be developed to further improve the densification and high-temperature structural stability. Finally, industrial scale-up preparation and on-site application tests will be conducted, combined with the actual float glass production process, so as to verify the engineering applicability and service performance of the novel tin bath bottom bricks.

## Figures and Tables

**Table 1:** Research program (wt. %).

Materials	1#	2#	3#	4#	5#	6#	7#
Flint clay 5–3 mm	34	20	16	12	34	34	34
Mullite 5–3 mm	0	14	18	22	0	0	0
Flint clay 3–1 mm	22	22	22	22	8	4	0
Mullite 3–1 mm	0	0	0	0	14	18	22
Flint clay 1–0 mm	13	13	13	13	13	13	13
High-alumina powder	22	22	22	22	22	22	22
α-Al_2_O_3_ micropowder	0–5	0–5	0–5	0–5	0–5	0–5	0–5
Silica fume	3–6	3–6	3–6	3–6	3–6	3–6	3–6
Calcium aluminate cement	3–6	3–6	3–6	3–6	3–6	3–6	3–6
Water reducer	0.15	0.15	0.15	0.15	0.15	0.15	0.15

**Table 2:** Reactions.

2CaO + Al_2_O_3_ + SiO_2_ = 2CaO·Al_2_O_3_·SiO_2_	(R1)
CaO + Al_2_O_3_ + 2SiO_2_ = CaO·Al_2_O_3_·2SiO_2_	(R2)
CaO + 6Al_2_O_3_ = CaO·6Al_2_O_3_	(R3)
CaO + Al_2_O_3_ = CaO·Al_2_O_3_	(R4)
2CaO + Al_2_O_3_ = 2CaO·Al_2_O_3_	(R5)
3Al_2_O_3_ + 2SiO_2_ = 3Al_2_O_3_·2SiO_2_	(R6)
Na_2_O + Al_2_O_3_ + 6SiO_2_ = Na_2_O·Al_2_O_3_·6SiO_2_	(R7)

**Table 3:** Apparent porosity (AP).

Mullite Addition	Mullite 5–3	Mullite 3–1
0%	18.06%	18.06%
14%	17%	17.22%
18%	16.5%	17.44%
22%	17.63%	17.86%

**Table 4:** Bulk density (BD).

Mullite Addition	Mullite 5–3	Mullite 3–1
0%	2.3 g/cm^3^	2.3 g/cm^3^
14%	2.39 g/cm^3^	2.37 g/cm^3^
18%	2.46 g/cm^3^	2.35 g/cm^3^
22%	2.32 g/cm^3^	2.31 g/cm^3^

**Table 5:** Cold crushing strength (CCS).

Mullite Addition	Mullite 5–3	Mullite 3–1
0%	50.26 Mpa	50.26 Mpa
14%	61.32 Mpa	59.63 Mpa
18%	69.9 Mpa	57.3 Mpa
22%	56.05 Mpa	56.32 Mpa

**Table 6:** Modulus of rupture (MOR).

Mullite Addition	Mullite 5–3	Mullite 3–1
0%	9.563 Mpa	9.563 Mpa
14%	11.331 Mpa	11.156 Mpa
18%	11.764 Mpa	10.89 Mpa
22%	11.289 Mpa	10.76 Mpa

**Table 7:** Refractoriness under load (RUL).

Mullite Addition	Mullite 5–3	Mullite 3–1
0%	1500 °C	1500 °C
14%	1539 °C	1529 °C
18%	1580 °C	1523 °C
22%	1519 °C	1520 °C

**Table 8:** Hydrogen diffusion.

Mullite Addition	Mullite 5–3	Mullite 3–1
0%	83 mmH_2_O	83 mmH_2_O
14%	73 mmH_2_O	74 mmH_2_O
18%	70 mmH_2_O	75 mmH_2_O
22%	76 mmH_2_O	77 mmH_2_O

**Table 9:** Mechanical resistance of sintered samples at high temperature.

Number of Cycles	Formula (R1)	Formula (R2)	Formula (R3)	Formula (R4)	Formula (R5)	Formula (R6)	Formula (R7)
0	9.563	11.331	11.764	11.289	11.156	10.89	10.76
1	7.346	9.864	10.745	10.23	9.81	8.77	8.56
3	7.018	9.433	10.486	9.943	9.36	8.54	8.06
5	6.885	9.218	10.137	9.596	9.22	8.16	7.97
10	4.263	6.564	7.311	6.892	7.43	5.16	4.83

## Data Availability

The original contributions presented in the study are included in the article; further inquiries can be directed to the corresponding authors.
